# Glutamate Transport and Preterm Brain Injury

**DOI:** 10.3389/fphys.2019.00417

**Published:** 2019-04-24

**Authors:** Silvia Pregnolato, Elavazhagan Chakkarapani, Anthony R. Isles, Karen Luyt

**Affiliations:** ^1^ Department of Neonatal Neurology, Translational Health Sciences, Bristol Medical School, University of Bristol, Bristol, United Kingdom; ^2^ Behavioural Genetics Group, MRC Centre for Neuropsychiatric Genetics and Genomics, School of Medicine, Cardiff University, Cardiff, United Kingdom

**Keywords:** preterm infant, brain injury, glutamate, excitotoxicity, inflammation, EAAT2, SLC1A2, GLT-1

## Abstract

Preterm birth complications are the leading cause of child death worldwide and a top global health priority. Among the survivors, the risk of life-long disabilities is high, including cerebral palsy and impairment of movement, cognition, and behavior. Understanding the molecular mechanisms of preterm brain injuries is at the core of future healthcare improvements. Glutamate excitotoxicity is a key mechanism in preterm brain injury, whereby the accumulation of extracellular glutamate damages the delicate immature oligodendrocytes and neurons, leading to the typical patterns of injury seen in the periventricular white matter. Glutamate excitotoxicity is thought to be induced by an interaction between environmental triggers of injury in the perinatal period, particularly cerebral hypoxia-ischemia and infection/inflammation, and developmental and genetic vulnerabilities. To avoid extracellular build-up of glutamate, the brain relies on rapid uptake by sodium-dependent glutamate transporters. Astrocytic excitatory amino acid transporter 2 (EAAT2) is responsible for up to 95% of glutamate clearance, and several lines of evidence suggest that it is essential for brain functioning. While in the adult EAAT2 is predominantly expressed by astrocytes, EAAT2 is transiently upregulated in the immature oligodendrocytes and selected neuronal populations during mid-late gestation, at the peak time for preterm brain injury. This developmental upregulation may interact with perinatal hypoxia-ischemia and infection/inflammation and contribute to the selective vulnerability of the immature oligodendrocytes and neurons in the preterm brain. Disruption of EAAT2 may involve not only altered expression but also impaired function with reversal of transport direction. Importantly, elevated EAAT2 levels have been found in the reactive astrocytes and macrophages of human infant post-mortem brains with severe white matter injury (cystic periventricular leukomalacia), potentially suggesting an adaptive mechanism against excitotoxicity. Interestingly, EAAT2 is suppressed in animal models of acute hypoxic-ischemic brain injury at term, pointing to an important and complex role in newborn brain injuries. Enhancement of EAAT2 expression and transport function is gathering attention as a potential therapeutic approach for a variety of adult disorders and awaits exploration in the context of the preterm brain injuries.

## Global Significance of Preterm Brain Injuries

Perinatal care has advanced considerably in the last century and has improved survival of many vulnerable newborns, including those born preterm. The World Health Organization estimates that 15 million newborns (1 in 10 live births) are born preterm (<37 weeks of gestation) worldwide each year (World Health Organization, 2012). Despite global improvements, the United Nations Millennium Development Goal to reduce childhood mortality by two-thirds in 2015 was not achieved globally (United Nations, 2015) and 2.7 million children died in the first month of life worldwide in 2015. Of these babies, over 900,000 died due to preterm birth complications – the leading cause of death of newborns and children under 5 years old (Liu et al., 2016). For the newborns who survive, the multi-organ damage can result in life-long disabilities. Globally, preterm birth complications represent the fourth leading cause of years of “healthy” life lost due to disability (i.e., over 102,000 DALYs), above causes such as diarrheal diseases, diabetes, and HIV (World Health Organization, 2016).

Prematurity is a major risk factor for cerebral palsy, “a group of permanent disorders of the development of movement and posture, causing activity limitation, that are attributed to non-progressive disturbances that occurred in the developing fetal or infant brain” (Bax et al., 2005; Rosenbaum et al., 2007). Cerebral palsy is the most common physical disability in childhood and is a heterogeneous diagnosis, including different clinical types and brain imaging patterns, comorbidities, and multiple causes (Stanley et al., 2000; Locatelli et al., 2010; MacLennan et al., 2015). Preterm birth is clearly an important risk factor and risk is 30 times higher in children born before 33 weeks of gestation than in those born at term (Stanley, 1992; Himpens et al., 2008; Beaino et al., 2010; Mercier et al., 2010; Tronnes et al., 2014; MacLennan et al., 2015; Stavsky et al., 2017). A recent meta-analysis estimated an increase in prevalence from 1.4/1,000 live births in children born at term (>36 weeks of gestation) to 6.8/1,000 live births in moderate to late preterm (32–36 weeks of gestation), rising to 43.2/1,000 live births in very preterm (28–31 weeks of gestation) and 82.3/1,000 live births in extremely preterm infants (<28 weeks of gestation) (Oskoui et al., 2013; Hirvonen et al., 2014). More than a third of the extremely preterm children with cerebral palsy are unable to walk (Moore et al., 2012), and many have multiple disabilities, which may further limit independence and quality of life (Litt et al., 2005; Glass et al., 2008, 2015; Soria-Pastor et al., 2008; Anderson et al., 2011; Moore et al., 2012). A systematic review of international cerebral palsy registers in high-income settings highlighted the extent of these comorbidities: around three quarters of children with cerebral palsy suffer from chronic pain; approximately half have intellectual disabilities (IQ, executive function, language ability); around a quarter have active epilepsy, hip dislocation, bladder control problems, behavioral problems, sleep disorders, and/or speech impairment; 11 and 4% have severe vision and hearing impairment, respectively (Novak et al., 2012). There are less data from low-income settings, but it is likely that comorbidities, as well as mortality, are higher (Khandaker et al., 2015). Preterm birth complications impose a considerable economic burden on the public sector, which was estimated around £2.9 billion in England and Wales in 2006 (Mangham et al., 2009). While administration of magnesium sulfate as a preventative treatment to the mother during preterm labor has been shown to reduce risk of cerebral palsy by a third in very preterm infants (Doyle et al., 2009), no postnatal therapy currently exists for preterm brain injury. This is a global health priority as the increase in both preterm birth and survival rates has not been matched by a decrease in long-term disability (Wilson-Costello et al., 2005).

## Neuroimaging and Neuropathology of Preterm Brain Injuries

Preterm birth is associated with smaller brain volumes (Peterson et al., 2003; Inder et al., 2005; Srinivasan et al., 2007) as well as motor, cognitive, and behavioral problems at school age (Peterson et al., 2000, 2003; Abernethy et al., 2004; Nosarti et al., 2005; Gimenez et al., 2006; Anderson and Doyle, 2008; Kesler et al., 2008; Aarnoudse-Moens et al., 2009; Delobel-Ayoub et al., 2009; Soria-Pastor et al., 2009; Anderson et al., 2017). Progress in neuroimaging techniques has been key in linking childhood neurodevelopmental outcomes to perinatal brain injuries and in advancing our knowledge of the underlying neuropathology (Volpe, 2009c; Back, 2017). Both MRI-defined preterm white matter injury (periventricular leukomalacia) and preterm birth are predictive of cerebral palsy (Constantinou et al., 2007; Spittle et al., 2008, 2009, 2018; Duerden et al., 2013). In a large European population study of cerebral palsy, white matter injury was the most common feature found in over 40% of the children (Bax et al., 2006). Originally, cranial ultrasound could only detect the most severe cystic type of white matter injury (cystic periventricular leukomalacia), characterized by focal macroscopic cysts of necrotic tissue in the deep white matter (de Vries et al., 1992) and highly predictive of cerebral palsy (Leviton and Paneth, 1990; De Vries et al., 2004; Serdaroglu et al., 2004; Fetters and Huang, 2007). Necrotic white matter injury can also evolve into microscopic glial scars, which may not be visible with traditional ultrasound. These are a more common type of injury and are sufficient to cause a loss in brain volume (Volpe, 2009c; Volpe et al., 2011). With the development of MRI techniques, a diffuse type of white matter injury has increasingly been recognized in the form of diffuse disturbances of myelination in the central white matter. This has emerged as the predominant type of white matter injury, accounting for over 90% of periventricular leukomalacia cases, as well as the predominant type of preterm brain injury altogether, occurring in 50% preterm newborns (Volpe, 2008). Importantly, while rates of the more severe cystic form have declined to less than 5% with advances in perinatal care, this has not been reflected for the diffuse forms (Maalouf et al., 2001; Counsell et al., 2003; Inder et al., 2003; Miller et al., 2003; Back et al., 2007b; Volpe, 2008). These could be seen as different manifestations of an “encephalopathy of prematurity” (Volpe, 2009c) or even as distinct pathologies (Back and Rosenberg, 2014). In the last two decades, advanced MRI techniques have highlighted that injury is not limited to the white matter but it extends to the deep grey matter, cortex, and cerebellum, all of which contribute to the volume loss (Counsell and Boardman, 2005; Ball et al., 2012). The cerebellum is gathering attention as a key target of injury. This region grows rapidly at the peak time for preterm birth and damage in the form of infarction, atrophy, and poor growth has been reported as common in very preterm infants developing cerebral palsy and long-term motor, cognitive, and behavioral impairment (Mercuri et al., 1997; Abraham et al., 2001; Bodensteiner and Johnsen, 2005; Johnsen et al., 2005; Limperopoulos et al., 2005a,b, 2007; Nosarti et al., 2008; Parker et al., 2008; Lawrence et al., 2014). Indeed, there is a relationship between cerebellar volume loss and white matter injury, pointing to the existence of a common insult, such as hypoxia-ischemia and infection/inflammation, which are known to damage the developing cerebellum (Shah et al., 2006; Volpe, 2009b; Hutton et al., 2014).

Disentangling the spatial and temporal contributions of infection/inflammation and hypoxia-ischemia will be key in understanding brain injuries across the perinatal spectrum. For example, while white matter injury is typical of the preterm newborn, it may be present in a subset of newborns born at term who experienced *in utero* hypoxic-ischemic insults (e.g., placental insufficiencies) (Mallard et al., 1998; Rees et al., 1998; Zhu et al., 2016). Indeed, newborns born at term with hypoxic-ischemic encephalopathy are also at high risk and up to 40% develop cerebral palsy (Gluckman et al., 2005; Shankaran et al., 2005; Azzopardi et al., 2009; Simbruner et al., 2010; Jacobs et al., 2011). Investigating the molecular basis for divergence between term and preterm injuries is paramount for development of age-appropriate pharmacological therapies.

## Pathogenesis of Preterm Brain Injuries

Brain injury is thought to be more common in preterm than term newborns for several reasons, including developmental and genetic vulnerabilities and differential exposure to adverse perinatal environments. A considerable body of *in vitro* and *in vivo* evidence points two potential triggers of injury, hypoxia-ischemia, and infection/inflammation (Volpe, 2008, 2009a; Deng, 2010; Volpe et al., 2011; Back and Rosenberg, 2014; Back, 2017). These insults are thought to interact in the vulnerable immature brain and converge onto three downstream mechanisms of injury: inflammation, glutamate excitotoxicity, and ultimately free radical attack, which directly damages cell components as well as triggering delayed cell death by apoptosis. Severity and temporal profile of hypoxia-ischemia and infection/inflammation, degree of brain maturity, comorbidities, sex, and genetic background may all contribute to individual differences in pathogenesis, clinical presentation, and individual susceptibility to injury. We will review the role of developmental vulnerabilities, infection/inflammation, and hypoxia-ischemia and bring the focus on the common downstream mechanism of glutamate excitotoxicity. We will then review the evidence linking glutamate transport to excitotoxic preterm brain injuries and highlight the current evidence supporting excitatory amino acid transporter 2 (EAAT2) as a potential therapeutic target.

### Developmental Vulnerability

The brain undergoes rapid and critical developmental events during the peak time of premature brain injury (24–32 weeks), including neuronal migration, growth of axons and dendrites, synaptogenesis, development of the vascular system, and myelination. Interference with these natural trajectories determines selective cellular and regional vulnerabilities and may redirect subsequent development. Among their functions, oligodendrocytes are responsible for laying the highly specialized myelin membrane around axons and are therefore key for the development of the white matter. Myelination begins before birth and peaks in the first 2 years of postnatal life, with the intracortical fibers of the cortex being myelinated in the third decade. The process of myelination requires that oligodendrocytes first proliferate and develop into mature oligodendrocytes and then depose myelin around axons (Volpe, 2008). Around the peak time of preterm brain injury (28–32 weeks of gestation), the pre-oligodendrocyte stage still represents the majority of the oligodendrial pool in the very preterm brain (Iida et al., 1995; Back et al., 2001). Pre-oligodendrocytes are more vulnerable than mature oligodendrocytes to hypoxia-ischemia, infection/inflammation, oxidative damage, and ultimately cell death (Back et al., 1998, 2002, 2005, 2007b; Fern and Moller, 2000; Baud et al., 2004; Fragoso et al., 2004; Segovia et al., 2008; Volpe et al., 2011). Indeed, a unique feature of periventricular white matter injury is an arrest in the development of oligodendrocytes at the pre-oligodendrocyte stage, leading to the abnormal myelination patterns typically seen through MRI (Back et al., 2007b; Volpe et al., 2011). More severe necrotic injury extends to all the cell components, leading to cysts and exacerbating myelin injury via focal axonal degeneration (Laptook, 2016; Back, 2017). Concurrent developmental vulnerabilities include the limited ability of the immature brain to synthesize appropriate amounts of growth factors needed for brain development and self-protection, and an immature immune system, potentially promoting an excessive and sustained inflammatory response (Gilles et al., 2018).

### Environmental Triggers of Injury: Hypoxia/Ischemia and Infection/Inflammation

Alongside the intrinsic developmental vulnerability of the immature brain, the preterm newborn is exposed to a range of potentially harmful exposures in the perinatal period. Supported by mounting experimental and epidemiological evidence, perinatal infection/inflammation leading to an overly intense inflammatory response, or a “cytokine storm”, has increasingly been recognized as a major risk factor not only for preterm birth but also for preterm white matter injury and long-term neurodisabilities (Yoon et al., 1996, 1997, 2000; Baud et al., 1999; Duggan et al., 2001; Dollner et al., 2002; Heep et al., 2003; Kaukola et al., 2004, 2006; Ellison et al., 2005; Bi et al., 2014). The preterm brain is often exposed to inflammation early during fetal development (e.g., maternal infections and chorioamnionitis) and usually for prolonged periods during postnatal life in the neonatal intensive care environment (e.g., neonatal infections, inflammatory comorbidities such as necrotizing enterocolitis), during critical phases of myelination and brain plasticity (Murphy et al., 1995; Grether and Nelson, 1997; Verma et al., 1997; Alexander et al., 1998; Dammann and Leviton, 1998, 2000, 2004; O’Shea et al., 1998; Leviton et al., 1999; Wu and Colford, 2000; Dammann et al., 2002; Rezaie and Dean, 2002; Stoll et al., 2002; Wu, 2002; Schlapbach et al., 2011; Hagberg et al., 2015; Anblagan et al., 2016). A combination of multiple inflammatory hits, antenatally and postnatally, has been shown to increase risk of brain injury and disability compared to single hits (Korzeniewski et al., 2014; van der Burg et al., 2016; Yanni et al., 2017). Indeed, pharmacological interventions targeting inflammation may have translational potential based on preclinical studies (Hagberg et al., 2015).

The role of hypoxia-ischemia in preterm brain injury is more controversial. In term newborns with hypoxic-ischemic encephalopathy, defined and acute hypoxic-ischemic events before or during birth (e.g., placental abruption, cord occlusion, and uterine rupture) are usually recognized by the clinician and represent the first step of a diagnosis of hypoxic-ischemic encephalopathy, aided by objective clinical and neuroimaging criteria. In the preterm newborn, a sentinel event is rarely recognized, and hypoxia-ischemia is generally assumed to have a more complex temporal profile, with intermittent or chronic nature (Laptook, 2016; Ohshima et al., 2016). However, it remains challenging to determine the individual contribution of hypoxia-ischemia among several coexistent factors, such as infection/inflammation, growth restriction, or hyperoxia (Gopagondanahalli et al., 2016). Physiologically, it is conceivable that the preterm brain is vulnerable to hypoxia-ischemia due to the anatomical and functional immaturity of the periventricular vasculature, which would make the periventricular white matter vulnerable to minor drops in cerebral perfusion (Takashima and Tanaka, 1978; Lou et al., 1979; De Reuck, 1984; Altman et al., 1988; Pryds, 1991; Miyawaki et al., 1998; Inage et al., 2000; Volpe, 2008; Laptook, 2016). The periventricular white matter has lower basal blood flow compared to grey matter regions in both humans (Greisen, 1986; Pryds et al., 1990) and the preterm fetal sheep (Szymonowicz et al., 1988; Gleason et al., 1989; Riddle et al., 2006). Further drops in blood flow are common in sick premature infants with respiratory disease due to lung immaturity (Soul et al., 2007). Mechanical ventilation may contribute to ischemia due to the vasoconstrictive effect of the induced cumulative hypocarbia (Shankaran et al., 2006). Perinatal hypoxic-ischemic episodes are also likely to play a key role, including ongoing placental pathologies, an overlapping risk factor for intrauterine growth restriction, low birthweight, and preterm birth. A meta-analysis recently reported an association between preterm brain injury and perinatal risk factors related to hypoxia-ischemia, including oligohydramnios, acidemia, low Apgar scores, apnea, respiratory distress syndrome, and seizures (Huang et al., 2017). However, the link between regional differences in blood flow and vulnerability to severe white matter injury is not consistent, and even in moderate ischemia, some regions of white matter are spared. This suggests that ischemia is necessary but not sufficient in isolation (Riddle et al., 2006; McClure et al., 2008; Back, 2017). Indeed, it has been suggested that more consistent evidence is needed to ascertain the specific role of hypoxic and ischemic events in preterm brain injury altogether and that future research should take into account contributions and interactions with other biological processes, including infection/inflammation and developmental vulnerability (Gilles et al., 2018). Importantly, the impact of hypoxia-ischemia on the cerebellum is also emerging, as shown by reports of volume loss and death of Purkinje cells and Bergmann glia in term newborns with hypoxic-ischemic encephalopathy and mid-late gestation fetal sheep exposed to asphyxia (Rees et al., 1997; Inage et al., 1998; Castillo-Melendez et al., 2004; Biran et al., 2012; Hutton et al., 2014). In an established mouse model of chronic hypoxia recapitulating perinatal brain injuries, damage to the cerebellum was reported in terms of a significant loss of GABAergic interneurons and a delay in dendritic arborization of Purkinje cells, followed by motor impairment and cerebellar learning deficits (Chahboune et al., 2009; Zonouzi et al., 2015; Sathyanesan et al., 2018).

Several experimental studies have shown that hypoxia-ischemia and infection/inflammation lead to worse brain and behavioral outcomes when they interact, and insults that are individually insufficient to cause injury can lead to injury when combined (Dommergues et al., 2000; Eklind et al., 2001; Lehnardt et al., 2003; Ikeda et al., 2004; Larouche et al., 2005; Favrais et al., 2007; Wang et al., 2007, 2009, 2010; Aden et al., 2010; van Tilborg et al., 2018). This has led to the multiple hit hypothesis of preterm brain injury, whereby a mild first event sensitizes the brain to subsequent insults (Leviton et al., 2013; Van Steenwinckel et al., 2014; Barnett et al., 2018). The current hypothesis is that hypoxia-ischemia triggers an inflammatory response *per se*. This additional endogenous response combined with the inflammation triggered by infection leads to a pro-inflammatory “cytokine storm,” which is not matched by upregulation of anti-inflammatory cytokines and neurotrophic factors. This in turn sensitizes the brain to hypoxic-ischemic injury by enhancing glutamate excitotoxicity and damaging the blood-brain barrier (Hagberg et al., 2015). Tertiary mechanisms of injury, mediated by epigenetic modifications, may sustain the sensitization in the long term and interfere with remodeling and repair mechanisms (Dammann, 2007; Fleiss and Gressens, 2012).

A substantial body of experimental evidence suggests that glutamate excitotoxicity triggered by hypoxia-ischemia and/or infection/inflammation plays a key role in the pathogenesis of preterm white matter injury (Hagberg et al., 2002; Johnston, 2005; Volpe, 2008; Deng, 2010; Volpe et al., 2011).

## Glutamate Excitotoxicity in the Preterm Brain

### Glutamate Homeostasis and Dysregulation

Glutamate is the main excitatory neurotransmitter in the mammalian brain (Meldrum, 2000). It is essential for brain function, orchestrating not only fast excitatory neurotransmission but also long-lasting neuronal changes necessary for memory, learning, and cognition. It is also fundamental during brain development, due to its role in regulating formation and elimination of synapses, as well as neuronal migration, proliferation, and viability. Glutamate is abundant inside the brain cells, and most neurons and glial cells have glutamate receptors distributed across most cellular elements, highlighting the importance of glutamatergic systems for normal function (Curtis and Johnston, 1974; Watkins and Evans, 1981; Bliss and Collingridge, 1993; Newcomer et al., 2000; Platt, 2007). Stimulation of a glutamatergic neuron results in Ca^2+^-dependent release of glutamate in the synapse by vesicular exocytosis. Extracellular glutamate binds to and activates post-synaptic ionotropic (NMDA, AMPA, and kainate receptors) and metabotropic (mGluR) glutamate receptors, stimulating the post-synaptic neurons *via* Ca^2+^ or Na^+^ influx and inducing intracellular signaling cascades that lead to physiological cellular responses, such as regulation of transcription factors and DNA replication (Nicholls and Attwell, 1990; Danbolt, 2001).

**Figure 1:**
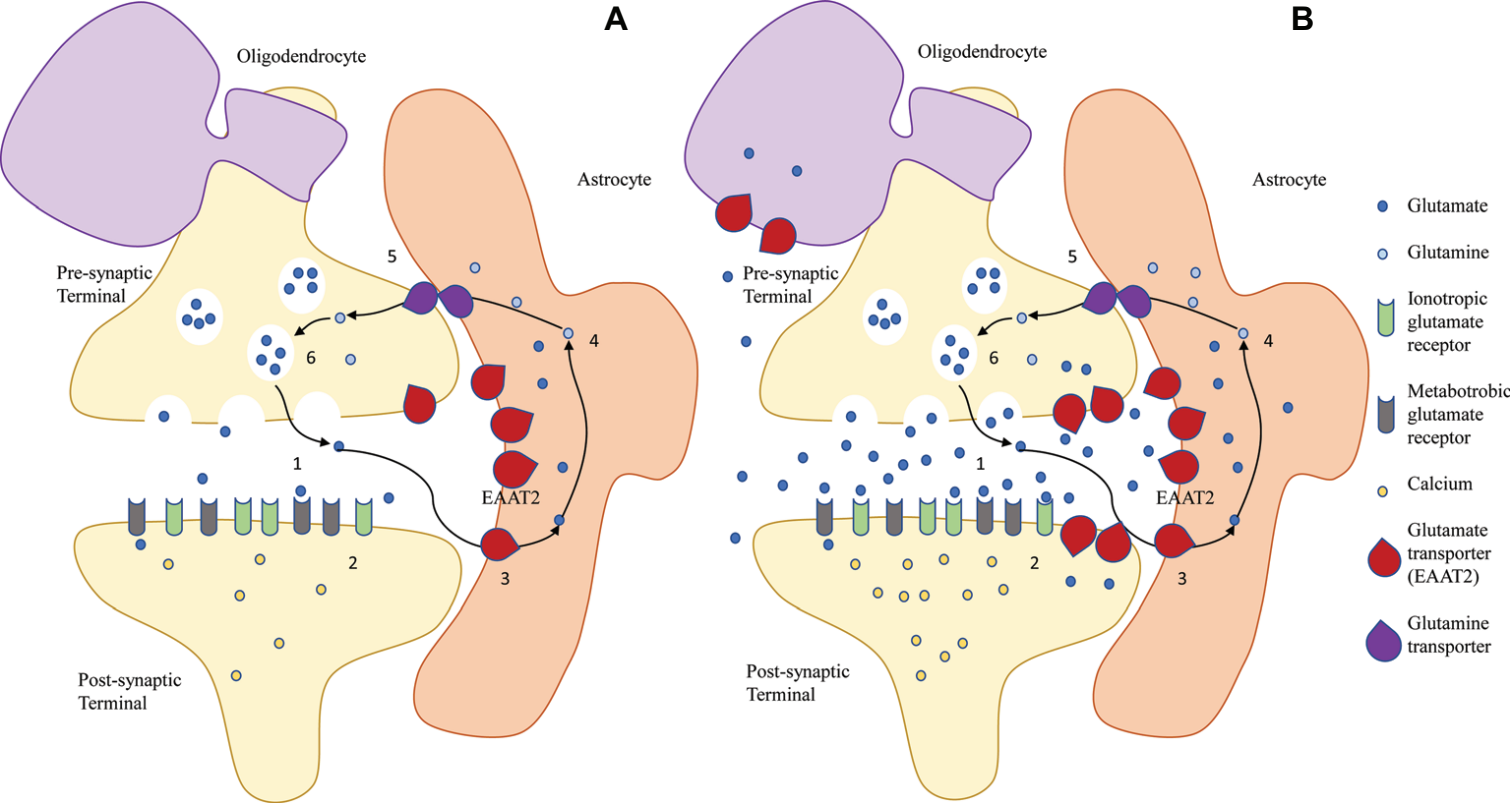
The glutamate/glutamine cycle in **(A)** physiological conditions and **(B)** excitotoxic conditions in the immature brain. **(A)** In the mature healthy brain, glutamate is released by exocytosis from the pre-synaptic neuronal terminal into the synapse (1), and it binds to post-synaptic ionotropic (NMDA, AMPA, and kainate receptors) and metabotropic (mGluR) glutamate receptors, inducing Ca^2+^-mediated signaling cascades that result in cellular responses (2). Extracellular glutamate is taken up primarily by astroglial EAAT2 (3) and converted to glutamine (4), which is shuttled back to the pre-synaptic terminal *via* glutamine transporters (5). Here, glutamine is converted back to glutamate (6). **(B)** During excitotoxicity, a combination of increased neuronal release and decreased astroglial uptake lead to a rise of extracellular glutamate levels, leading to overactivation of the post-synaptic glutamate receptors, Ca^2+^ overload, and activation of apoptotic pathways. Reversal of transport of astroglial transporters may also contribute to the accumulation of extracellular glutamate. In the immature brain, upregulation of the glutamate transporters in underdeveloped neurons and oligodendrocytes may contribute to their selective vulnerability.

Glutamatergic transmission is terminated when glutamate transporters, expressed predominantly by astrocytes, slowly take up glutamate from the synaptic space (30 glutamate molecules per second at Vmax) (Otis and Kavanaugh, 2000; Bergles et al., 2002; Grewer and Rauen, 2005; Takahashi et al., 2015). In the preterm brain, glutamate transporters are also expressed by immature neurons and oligodendrocytes, although their significance is controversial, as reviewed below. In astrocytes, glutamate is converted to glutamine *via* glutamine synthetase. Glutamine is shuttled back into the pre-synaptic neuron, where it is converted into glutamate *via* glutaminase (Figure 1). The glutamate-glutamine cycle is not essential for supplying glutamate for neuronal release but is needed for normal glutamatergic transmission (Danbolt, 2001; Takahashi et al., 2015; Danbolt et al., 2016).

The ubiquity of glutamate is a double-edged sword: when homeostasis is disrupted, glutamate can turn into a potent neurotoxin. If the concentration of glutamate in the extracellular space rises above physiological levels, post-synaptic glutamate receptors are overactivated. This excessive activation, or excitotoxicity, leads to cell death *via* activation of suicide cell programs (apoptosis) (Danbolt, 2001; Sattler and Tymianski, 2001) (Figure 1). Since it was first proposed in the late 1960s (Olney, 1969), the concept of glutamate excitotoxicity has been implicated in several adult disorders, both acute (e.g., ischemic stroke and traumatic brain injury) and chronic (e.g., amyotrophic lateral sclerosis, Alzheimer’s, Parkinson’s, major depression, and addiction) (Doble, 1999; Takahashi et al., 2015). Consistently, injection of glutamate agonists into the cortex, striatum, and periventricular white matter of newborn rodents, rabbits, and kittens produces patterns of perinatal brain injuries similar to those seen in humans (McDonald et al., 1988; Innocenti and Berbel, 1991a,b; Marret et al., 1995; Gressens et al., 1996; Acarin et al., 1999; Follett et al., 2000). On the other hand, pharmacological inhibition of glutamate receptors before or immediately after an hypoxic-ischemic insult is neuroprotective in both preterm (Follett et al., 2004; Manning et al., 2008) and term (Hagberg et al., 1994; Follett et al., 2000) brain injuries. Indeed, one of the mechanisms through which magnesium sulfate is thought to exert neuroprotection is by preventing excitotoxic damage through NMDA receptor blockade (Lingam and Robertson, 2018).

### *In vivo* Evidence of Glutamate Excitotoxicity

Evidence of *in vivo* disturbance of glutamate signaling has been produced for animal models of hypoxic-ischemic brain injury. In a rat model of mild white matter injury near term, a rise in extracellular glutamate is observed in the acute phase after hypoxia-ischemia, with oligodendrocytes and axons representing the major sources of extracellular glutamate and astrocytes failing to take up excess glutamate (Back et al., 2007a). Similarly, repeated umbilical cord occlusion in the near-term fetal sheep causes periventricular white matter injury, the extent of which correlates with extracellular local glutamate levels (Loeliger et al., 2003). Notably, the largest increase in glutamate occurred over the hours after the insult, a delayed increase that suggested impaired glutamate transport. In a piglet model of hypoxic-ischemic encephalopathy at term, glutamate levels in the basal ganglia were shown to change in two phases: an early increase in the first 6 hours was followed by transient and slight recovery by 12 hours, possibly due to the self-protective glutamate transport mechanisms and conversion to glutamine in astrocytes; a further increase occurred after a day, possibly through cells bursting due to reperfusion injury and reversal of glutamate transport in the late stages of disease (Dang et al., 2017). In humans, elevated glutamate levels have been reported in the cerebrospinal fluid and basal ganglia of asphyxiated newborns (Riikonen et al., 1992; Hagberg et al., 1993). Moreover, elevated glutamine levels have been found in MRI-defined punctate necrotic white matter lesions (Wisnowski et al., 2013). Glutamate is taken up into astrocytes for conversion into glutamine and shuttling back to neurons. The finding of elevated glutamine rather than glutamate may be due at least in part to the temporal lag between insult and measurement. An important limitation of *in vivo* glutamate measurements in preterm newborns is that the peak window of glutamate changes is probably missed, because magnetic resonance measurements are likely to be carried out long after the initial insults in newborns that have already become sick. As such, these findings suggest that disrupted glutamate homeostasis persists in the subacute phase in moderate necrotic white matter injury. Although a relatively small subset of the newborns with punctate lesions also had evidence of cysts, no studies to date have measured glutamatergic metabolism specifically in newborns with severe cystic white matter injury.

### Glutamate Excitotoxicity Following Hypoxia-Ischemia

Glutamate homeostasis can be disrupted by an acute hypoxic-ischemic event, and the phases of the subsequent excitotoxic injury are well described. During the primary energy failure, oxygen and blood deprivation lead to impairment of ATP production due to failure of oxidative phosphorylation. Astrocytes, with their unique oxidative capacity and ability to upregulate ATP production, are central to maintaining energy metabolism during the first stage of ischemia (Dienel and Hertz, 2005). Impairment of the ATP-dependent Na^+^/K^+^ pumps leads to loss of the electrochemical gradient across the cell membrane. If the insult is severe, some cells may die at this early stage *via* necrosis, due to influx of ions and water, cell swelling, and bursting. Within hours, the necrotic injury due to severe energy failure leads to death of all cellular elements and develops into the white matter cysts (Back, 2017). Depolarization of the cell membrane activates Ca^2+^ channels in the pre-synaptic terminal, triggering vesicular release of glutamate in the synapse. In astrocytes, hypoxia-ischemia leads to a failure in the astrocytic glutamate uptake system, which also relies on Na^+^/K^+^ gradients. The combination of increased synaptic release and reduced astrocytic uptake leads to accumulation of glutamate in the synaptic space and overactivation of post-synaptic ionotropic and metabotropic glutamate receptors (Volpe, 2008). The subsequent intracellular Ca^2+^ influx triggers activation of phospholipases, endonucleases, proteases, and nitric oxide synthase, with degradation of cellular and extracellular structures, and generation of harmful free radicals and reactive oxygen and nitrogen species. Glutamate leaking outside the synapse activates extrasynaptic NMDA receptors, which, contrarily to the pro-survival action of synaptic NMDA receptors, promotes excitotoxic cell death even further (Parsons and Raymond, 2014). This excitotoxic-oxidative cascade eventually leads to cell damage or death *via* necrosis, apoptosis, and autophagy in the secondary phase of injury (Olney, 1969; Benveniste et al., 1984; McDonald and Johnston, 1990; Choi, 1992; Thornton et al., 2012; Back, 2017; Descloux et al., 2018) (Figure 1).

### Glutamate Excitotoxicity Following Inflammation

In preterm brain injury, comorbidities stimulating inflammation are thought to contribute to disruption of glutamate homeostasis and potentiation of excitotoxicity. TNFα, for example, is one of the most studied cytokines and is emerging as a key link between inflammation and glutamate excitotoxicity (Olmos and Llado, 2014). TNFα has both neuroprotective and neurotoxic effects depending on the different signaling pathways activated by the different receptors. In fact, pharmacological inhibition or genetic deletion after a combined inflammatory and excitotoxic insult is neuroprotective (Aden et al., 2010; Kendall et al., 2011), but knocking out TNFα receptors in the mouse increases susceptibility to hypoxic-ischemic injury (Bruce et al., 1996). TNFα potentiates glutamate excitotoxicity *in vitro via* complex and interacting mechanisms involving crosstalk between neurons and glial cells and leading to vicious cycles of glutamate and cytokine release. In neurons, TNFα increases the excitatory strength at the synapse by increasing cell surface expression of glutamate receptors and their permeability to Ca^2+^, while also decreasing expression of inhibitory GABA_A_ receptors (Olmos and Llado, 2014). In microglia, TNFα stimulates autocrine release of TNFα and glutamate by upregulating glutaminase and from hemichannels of gap junctions (Takeuchi et al., 2006). In astrocytes, TNFα stimulates glutamate release *via* prostaglandin E2 and exacerbates impairment of glutamate transport (Bezzi et al., 1998). Cheung et al. (1998) suggested that glutamate concentration may be key in determining the pathways of cell death, with higher glutamate concentrations preferentially triggering necrosis and lower concentrations leading to apoptosis. Either way, even transient excess of glutamate can start a number of events that ultimately cause death or damage of vulnerable cell populations (Ottersen et al., 1996).

### Glutamate Excitotoxicity and Perinatal Brain Injuries

The patterns of excitotoxic injury tend to be different in the preterm and term brain. Experimental evidence suggests that the main cellular target of excitotoxic injury in the preterm brain is pre-oligodendrocytes (Volpe et al., 2011). Glutamate is highly toxic to pre-oligodendrocytes in cell culture and leads to cell death *via* free radical attack (Oka et al., 1993). The white matter in the rat is much more vulnerable to hypoxia-ischemia at preterm-equivalent age, when pre-oligodendrocytes are predominant, than at term-equivalent age, when mature oligodendrocytes are the major form (Back et al., 2002; Craig et al., 2003; Dean et al., 2011). Indeed, the patterns of hypoxic-ischemic white matter injury seem to be determined primarily by the timing of appearance (Buser et al., 2010) and spatial distribution (Riddle et al., 2006) of pre-oligodendrocytes rather than severity of ischemia itself. Pre-oligodendrocytes are strikingly more vulnerable than immature neurons of the cortex and caudate nucleus in moderate global ischemia in the preterm fetal sheep (Dean et al., 2013; McClendon et al., 2014). Immature neurons are also vulnerable, as NMDA receptors are functionally upregulated, more permeable to calcium and less sensitive to magnesium block (Jantzie et al., 2015).

In physiological conditions, the abundance of glutamate receptors in the white matter is key during early neuronal development, contributing to rapid growth and myelination. However, their abundance also confers increased vulnerability in excitotoxic conditions (Kaindl et al., 2009). Indeed, the selective vulnerability of subplate neurons compared to cortical neurons observed in a preterm model of hypoxia-ischemia has been suggested to originate from an increase of glutamate receptors in these neurons associated with early maturation (McQuillen et al., 2003). Similarly, it has been suggested that selective vulnerability of the deep grey matter and sensorimotor cortex in term hypoxic-ischemic encephalopathy could be related to peaking NMDA receptor expression and proximity to developing glutamatergic circuits (Rocha-Ferreira and Hristova, 2016). As such, developmental expression of key glutamatergic genes in the grey and white matter may contribute to the different patterns of excitotoxic injury (Volpe, 2008).

Overall, the potential sources of extracellular glutamate in the white matter include pre-oligodendrocytes, astrocytes, neurons, ependymal cells, and cells of the choroid plexus (Back and Rosenberg, 2014). While therapies targeting excitotoxicity have so far mostly focused on glutamate receptor blockade, targeting glutamate transport is gathering interest as a potential avenue for neuroprotection by counteracting glutamate accumulation in the first place (Tilleux and Hermans, 2007; Kim et al., 2011; Fontana, 2015; Takahashi et al., 2015).

## Glutamate Transport: Focus on EAAT2/GLT-1

Maintaining the baseline extracellular glutamate concentrations in the nanomolar range is essential to avoid extracellular glutamate build-up. The brain has no known enzymatic mechanism to metabolize glutamate in the extracellular space, and simple diffusion over short distances is thought to bring only a minor contribution. Hence, the brain relies substantially on intracellular glutamate uptake, and astrocytes provide by far the largest contribution to preventing excitotoxicity through expression of glutamate transporters (Danbolt, 2001; Tzingounis and Wadiche, 2007; Vandenberg and Ryan, 2013). Given their crucial role, it is not surprising that expression of astrocytic glutamate transporters is constitutively high (Zhou and Danbolt, 2013). Crosstalk between neurons and glia relies on tightly controlled extracellular glutamate homeostasis, and it is becoming increasingly evident that neuron-glia interactions are central to both the kinetics of glutamatergic synaptic activity in physiological (Fontana, 2015) and excitotoxic conditions (Carmignoto, 2000). Glutamate is released by astrocytes in immature rat optic nerve in ischemia *in vitro* (Wilke et al., 2004). Moreover, glutamate transport has been observed in immature axons (Arranz et al., 2008), and impairment has been reported in pre-oligodendrocytes during hypoxia-ischemia, providing a potential mechanism of excitotoxic vulnerability (Oka et al., 1993; Domercq et al., 1999; Fern and Moller, 2000; Deng et al., 2003; Desilva et al., 2007, 2009). The importance of glutamate transport to the integrity of oligodendrocytes and white matter is supported by evidence of extensive excitotoxic injury in oligodendrocytes and axons with experimental inhibition of glutamate transport in the optic nerve *in vivo* (Domercq et al., 2005).

The five members of the excitatory amino acid transporter (EAAT) family carry out most of the glutamate clearance in the central nervous system (Anderson and Swanson, 2000), especially EAAT1 (SLC1A3, rodent orthologue Glast) and EAAT2 (SLC1A2, rodent orthologue Glt-1) (Bristol and Rothstein, 1996). EAAT2 is the major glutamate transporter in the forebrain, except in the cerebellum, circumventricular organs, and retina, where EAAT1 is prevalent. In physiological conditions, both EAAT1 and EAAT2 are expressed predominantly by astrocytes and localized to the cellular membrane in the adult brain (Danbolt, 2001; Roberts et al., 2014; Takahashi et al., 2015). The high concentration (1 mg/g rat brain tissue), ubiquity (1% of total CNS protein in the adult brain), and high degree of conservation across mammalian species are all indications of physiological importance of EAAT2/Glt-1 (Danbolt, 2001; Fontana, 2015; Danbolt et al., 2016). Unsurprisingly, it is expressed at high density near glutamatergic synapses in developing hippocampal astrocytes, with density and vicinity increasing with neuronal activity (Benediktsson et al., 2012). This transmembrane transporter carries out glutamate uptake through a high affinity energy-dependent process driven by Na^+^ and K^+^ gradients. Specifically, glutamate and aspartate are co-transported inside the brain cells with 3 Na^+^ and 1 H^+^ for the antiport of 1 K^+^. EAAT2 is also a selective anion channel, transporting Cl^−^ anions during intermediate conformations, uncoupled from the flux of glutamate (Fontana, 2015).

Several lines of evidence support the central role of EAAT2 expression/function in maintaining extracellular glutamate homeostasis. Pharmacological inhibition of glutamate transport, including EAAT2, leads to rapid extracellular glutamate increase *in vitro* (Jabaudon et al., 1999) and extended post-synaptic activation mediated by NMDA receptors (Lozovaya et al., 1999). Genetic deletion of Glt-1 *via* constitutive knockout in the mouse leads to lower body weight, seizures, acute cortical injury in the forebrain, and increased mortality from the second/third postnatal week (Tanaka et al., 1997). Brain tissue from this mouse shows much lower (5%) glutamate transport activity than wild-type, suggesting that Glt-1 is responsible for up to 95% of glutamate transport. This is confirmed by the ability of Glt-1 antibodies to remove 90% of the transport activity in forebrain tissue (Haugeto et al., 1996). Other Glt-1 knockouts have confirmed the obvious phenotype, with lower life span, lower body and brain weight, mild loss of CA1 neurons in the hippocampus, and severe focal neuronal loss in layer II of the neocortex and focal gliosis (Kiryk et al., 2008). A conditional knockout mouse with selective deletion of Glt-1 reproduces this phenotype while ruling out developmental adaptations (Zhou et al., 2014). Heterozygote knockouts, on the other hand, show halved concentrations of Glt-1, but no apparent morphological brain changes, despite an increased risk of traumatic spinal cord injury (Kiryk et al., 2008; Lepore et al., 2011). Inhibition with antisense oligonucleotides *in vitro* and *in vivo* induces a rise in extracellular glutamate, excitotoxic injury, and progressive paralysis (Rothstein et al., 1996). On the other hand, selective overexpression in astrocytes is neuroprotective during ischemia (Chao et al., 2010).

Studies of EAAT2 expression point to different patterns depending on cell type, region, developmental age, species, and methodology used (DeSilva et al., 2012). In the adult rat, Glt-1 is expressed in the forebrain, especially in the hippocampus, cortex, striatum, and thalamus as well as in fibrous astrocytes in the white matter (Lehre et al., 1995). The transporter is expressed predominantly by astrocytes but also pre-synaptic axon terminals in the rodent hippocampus and somatosensory cortex (Danbolt, 2001; Chen et al., 2004; Furness et al., 2008; Melone et al., 2009; de Vivo et al., 2010; Danbolt et al., 2016). Neuronal EAAT2 represents no more than 10–20% total EAAT2 (Furness et al., 2008; Danbolt et al., 2016), and while being implicated in adult neuropsychiatric disorders (O’Donovan et al., 2017), neuronal knockout barely affects total Glt-1 protein levels and mouse development (Petr et al., 2015). Conversely, astrocytic knockout leads to a reduction of protein levels to a fifth in the forebrain, lower body weight and increased epilepsy and mortality.

### Developmental Expression of EAAT2

The scenario may be at least in part different in the preterm brain, where transient but more prominent neuronal and pre-oligodendrial expression is observed. During development, dynamic and species-specific changes in both cellular and regional expression have been observed, suggesting that glutamate transporters may be both regulated by and involved in brain development (e.g., participation in the development of the topographic organization). As expected, these changes in rodent Glt-1 expression correspond to changes in total glutamate uptake activity (Ullensvang et al., 1997). Briefly, Glt-1 expression is low until after birth, except for a transient peak of expression in developing axons and oligodendrocytes around mid-late gestation. Glt-1 is expressed *in vivo* in rat pre-oligodendrocytes, whereas it is no longer detectable in mature oligodendrocytes (DeSilva et al., 2009). Transient neuronal expression is also seen around mid-late gestation in the mouse (Sutherland et al., 1996; Yamada et al., 1998), rat (Furuta et al., 1997), and sheep (Northington et al., 1998). In the fetal rat, Glt-1 is expressed in the amygdala and hippocampus, as well as white matter tracts interconnecting neocortex, basal ganglia, and thalamus (Furuta et al., 1997). In the fetal sheep, Glt-1 is found not only in white matter tracts but also in neuronal bodies and extended to the subplate, cranial nerve nuclei, basal ganglia, and cerebellar cortex, highlighting potential species differences in cellular expression during development (Furuta et al., 1997; Northington et al., 1998, 1999). In the newborn rat at P1, Glt-1 levels are the highest in the spinal cord and moderate in the hippocampus and hypothalamus. Expression increases dramatically from the second postnatal week throughout the central nervous system, especially in the cortex, striatum, caudate nucleus, and hippocampus, reaching adult levels by weeks 4–5 (Rothstein et al., 1994; Levy et al., 1995; Shibata et al., 1996; Sutherland et al., 1996; Furuta et al., 1997; Ullensvang et al., 1997). Astrocyte selectivity is established in the postnatal period in rodents and around mid-late gestation in sheep (Furuta et al., 1997; Takasaki et al., 2008). Nonetheless, Glt-1 is still detected in immature axons at P14–17 (Arranz et al., 2008). The significant developmental changes in Glt-1 after birth may explain why the Glt-1 knockout mice seem to develop normally for the first few weeks and develop seizures and brain injury during postnatal week 3, with many dying by week 4 (Tanaka et al., 1997; Takasaki et al., 2008).

A limited number of studies have investigated developmental regulation of EAAT2 in humans. DeSilva et al. (2012) found that, among EAAT1–3, expression of EAAT2 undergoes particularly prominent maturational changes in post-mortem cortex tissue of preterm and term newborns without neurological disease, all the way into childhood. Consistent with animal studies, EAAT2 expression is generally low until birth and is limited to glia limitans, layer I-III fine astrocytes, and some neuron populations. EAAT2 was found not only in axons but also in the cell body and dendrites of certain neuron populations from as early as 23 gestational weeks up until term and, in some cases, until 8 postnatal months. These neuron populations are layer V pyramidal neurons, layer I neurons (putative Cajal-Retzius cells), and subplate neurons (DeSilva et al., 2012). A great proportion of these neuronal populations is glutamatergic, and it has been suggested that this transient neuronal EAAT2 expression is critical for establishing and orchestrating excitatory transmission during maturation and migration of cortical neurons. Similarly, it could also provide the basis for selective vulnerability to premature excitotoxic injury due to expression of glutamate transporters, which may reverse transport and become sources of extracellular glutamate (Takasaki et al., 2008; DeSilva et al., 2012), as discussed below. This is supported by evidence of selective vulnerability of layer V pyramidal neurons and subplate neurons in human and rat preterm white matter injury (McQuillen et al., 2003; Andiman et al., 2010). The same group reported EAAT2 expression in pre-oligodendrocytes in human fetal white matter at 32 weeks of gestation, during the peak time for premature brain injury, but not at 7 months old, consistent with rat studies (Desilva et al., 2007). EAAT2 expression appeared in the astrocytes of the developing cortex at 41 postconceptional weeks, increasing steeply in the first 1.5 years (DeSilva et al., 2012). Taken together, these findings suggest that the expression of EAAT2/Glt-1 undergoes substantial changes during development and that these changes may contribute to the selective vulnerability of cellular (e.g., immature oligodendrocytes and neurons) and regional (e.g., white matter tracts, hippocampus) targets in preterm brain injury.

### EAAT2 and Preterm Brain Injury

Following severe energy failure, the dissipation of the transmembrane gradient impairs astrocytic EAAT2, which relies on transmembrane Na^+^/K^+^ gradients. This disruption may involve both quantity and quality of transport activity, i.e., it can manifest as decreased expression and/or impairment of glutamate transport function with establishment of reverse transport. Reverse transport has an outward direction and is driven by the transmembrane gradient of excitatory amino acids independently of ATP and Ca^2+^ (Nicholls and Attwell, 1990; Szatkowski et al., 1990; Levi and Raiteri, 1993). In this scenario, glutamate transporters become themselves a major source of extracellular glutamate, potentially turning into key contributors of excitotoxic injury in any cells expressing them (Domingues et al., 2010) (Figure 1). While its significance to preterm brain injuries remains to be explored, the importance of reverse transport is supported by evidence that ischemic cell death in the rat striatum can be blocked by an inhibitor of reverse Glt-1 transport (Seki et al., 1999). Moreover, knockout mice lacking Glt-1 are more vulnerable to neuronal death after a short, severe episode of ischemia than wild-type mice, suggesting that Glt-1 is essential for neuroprotection when ischemia is acute; on the other hand, wild-type mice expressing Glt-1 are more vulnerable to neuronal death than mice lacking Glt-1 during extended, chronic ischemia, suggesting that Glt-1 (*via* reverse transport) becomes neurotoxic when ischemia is prolonged (Mitani and Tanaka, 2003).

Consistent with impairment of glutamate transport, a decrease in glutamate uptake is seen in the hippocampus of rat pups exposed to intrauterine hypoxia following caesarean delivery (Frizzo et al., 2010) and in the cortex, basal ganglia and thalamus of newborn piglets exposed to hypoxia (Jantzie et al., 2010). Loss of Glt-1 expression and/or function has been reported in astrocyte cultures during hypoxia (Dallas et al., 2007) as well as in the adult rat cortex and hippocampus after ischemia (Torp et al., 1995; Rao et al., 2001a,b). In a small study of term-equivalent rats, astrocytic Glt-1 was suppressed in the initial 12 hours in the ischemic core of both the hippocampus and the neocortex, recovered after 48 hours only in the hippocampus, followed by astrogliosis at 72 hours (Fukamachi et al., 2001). In a piglet model of hypoxic-ischemic encephalopathy at term, canonical suppression of Glt-1 in astrocytes of the striatum and hippocampus was accompanied by upregulation in neurons of the striatum (Martin et al., 1997b; Danbolt, 2001; Pow et al., 2004; Desilva et al., 2007, 2012). The striatum is known to be selectively vulnerable to excitotoxicity at term, and this may suggest a potential neuronal response to locally increasing extracellular glutamate levels (Martin et al., 1997a). In P6 rats, exposure to hypoxic preconditioning led to upregulation of Glt-1 in the cortex and suppression in the striatum, with no detectable changes in the hippocampus (Cimarosti et al., 2005). Glt-1 was also suppressed in the white matter in a preterm mouse model of chronic hypoxia, although this model was not subjected to ischemia and showed no sign of reactive astrogliosis (Raymond et al., 2011). Moreover, hypoxia has been found to alter the expression of Glt-1 splice variants in mouse brain and neurons of newborn pigs (Munch et al., 2003; Pow et al., 2004).

Exposure of mouse astrocytes, rat microglia, and human blood macrophages to the bacterial endotoxin lipopolysaccharide (LPS) and the pro-inflammatory cytokine TNFα has been found to enhance EAAT2 expression and glutamate uptake function *in vitro* (Rimaniol et al., 2000; Persson et al., 2005; O’Shea et al., 2006). On the other hand, TNFα suppresses both glutamate uptake and EAAT2 in a dose-dependent manner (via NF-κB) in human fetal astrocytes (Fine et al., 1996; Liao and Chen, 2001; Su et al., 2003). TNFα also selectively suppresses EAAT2 *via* NF-κB during hypoxia *in vitro* (Boycott et al., 2008).

An important finding is that EAAT2 is upregulated in the reactive astrocytes and macrophages of post-mortem human brain tissue from preterm babies with white matter injury compared to controls, suggesting a possible response to hypoxia-ischemia and/or inflammation in the preterm brain (Desilva et al., 2008). Pre-oligodendrocytes in both cases and controls expressed EAAT2, with no qualitative differences in expression, although function was not measured. Upregulation of EAAT2 in reactive astrocytes and macrophages in preterm white matter injury may be an adaptive mechanism to counteract excitotoxicity, or it could be a secondary mechanism due to gliosis. Whether in chronic white matter injury, this upregulation contributed to excitotoxicity *via* transport reversal remains to be established. Further studies are needed to elucidate how perinatal hypoxia-ischemia and infection/inflammation affect EAAT2 homeostasis, separately and in combination. Interestingly, genome-wide gene expression analysis of reactive astrocytes in two adult mouse models of ischemic stroke and LPS-induced neuroinflammation revealed that at least half of the altered gene expression is specific on the insult, with indication that reactive astrocytes may be neuroprotective in ischemia but detrimental in neuroinflammation (Zamanian et al., 2012). Overall, candidacy of EAAT2 is supported by the fact that dysregulation is implicated in several neurological, neurodegenerative, and psychiatric disorders thought to involve glutamate excitotoxicity (i.e., transient cerebral ischemia, ischemic stroke, epilepsy, traumatic brain injury, amyotrophic lateral sclerosis, Alzheimer’s disease, Parkinson’s disease, chronic pain, Huntington’s disease, HIV-associated cognitive disorder, glioma, major depression, schizophrenia, and addiction) (Danbolt, 2001; Beart and O’Shea, 2007; Fontana, 2015; Karki et al., 2015; Takahashi et al., 2015; Verkhratsky et al., 2016; Zhang et al., 2016; Zhou et al., 2016; Goodwani et al., 2017; O’Donovan et al., 2017; Assefa et al., 2018; Fogarty, 2018; Kim et al., 2018; Parkin et al., 2018).

A better understanding of the role of glutamate transport in preterm brain injuries will require further investigations of EAAT1 in the cerebellum. EAAT1 is highly expressed in cerebellar astrocytes, particularly Bergmann’s glia (Lehre et al., 1995; Danbolt, 2001). The processes of these cells ensheath the Purkinje cell synapses, which have been suggested to be selectively vulnerable to excitotoxicity induced by hypoxia-ischemia (Harding et al., 1984; Shibata et al., 1996). Indeed, EAAT1 is developmentally upregulated from 23 weeks gestation, possibly in conjunction with the maturation of the Purkinje cells. Importantly, EAAT1 undergoes rapid changes in hypoxic-ischemic encephalopathy at term, with a decrease in the molecular layer and an increase in the Purkinje and inner granule cell layer at an early stage. This increase becomes marked at a later stage, potentially pointing to an adaptive neuroprotective mechanism against excitotoxicity (Inage et al., 1998).

Mechanisms leading to loss of expression and/or function are likely to be complex. Ying’s (1997) “deleterious network hypothesis” (1997) suggests that glutamate build-up may lead to detrimental vicious cycles. For example, receptor overactivation may lead to increased energy consumption and oxidative damage, which is known to impair glutamate transporters’ activity and expression, potentially leading to reverse transport with further glutamate release. Ion flux may cause cell swelling, leading to impaired energy metabolism (Danbolt, 2001). Inflammation may further potentiate the risks of excitotoxicity *via* glutamate transport suppression, including selective effects on EAAT2 (Aden et al., 2010; Kapitanovic Vidak et al., 2012). Evidence to date supports the concept of suicide loops in pre-oligodendrocytes, which could provide both the source and the target for excitotoxic injury in the preterm brain. In this context, the combination of developmental upregulation of EAAT2 and establishment of reverse transport in the context of an energy failure could increase vulnerability of pre-oligodendrocytes to excitotoxic death (Back and Rosenberg, 2014). Similarly, transient expression in neuronal populations could feed into suicide loops and explain the loss of layer V pyramidal neurons accompanying necrotic PVL (Andiman et al., 2010). This is a different mechanism to that hypothesized in the mature brain, where the sources of glutamate killing neurons are thought to be other cells, including astrocytes and excitatory terminals (Lipton and Rosenberg, 1994) or, alternatively, retrograde degeneration from axonal injury. Astrocytes may have a delayed response due to their unique ability to use glycogen as a metabolic fuel during the initial stages of energy deprivation. In this scenario, extracellular glutamate concentrations may rise significantly only after depletion of glycogen stores in astrocytes (Grewer et al., 2008), with a subsequent steep rise in extracellular glutamate and excitotoxic cell death (Gouix et al., 2009). In chronic white matter injury, upregulation of astrocytic EAAT2 may be detrimental when accompanied by establishment of reverse transport. Experimental data are needed to evaluate these hypotheses.

## Potential Future Developments

In summary, it is plausible that both up- and downregulation of EAAT2 contribute to disease, depending on animal model, developmental stage, type and severity of the insult, and comorbidities. Regulation and dysregulation of EAAT2 may occur at the level of transcription (including epigenetic regulation), translation, trafficking, transport, and degradation (Karki et al., 2015; Takahashi et al., 2015). Accordingly, treatments aiming at restoring EAAT2 expression are a current area of research in neuroprotection, alongside enhancement of the transport function (Fontana, 2015). Ceftriaxone, a licensed β-lactam antibiotic safe and tolerable for humans, enhances EAAT2 expression and has been shown to be neuroprotective in animal models of several adult excitotoxic disorders. Although no significant effects have been seen in clinical trials for amyotrophic lateral sclerosis and adult stroke, it is already widely used for the treatment of CNS infections in newborns and would therefore be a feasible drug to explore in the context of preterm neuroprotection. Guanosine enhances EAAT2 transport function and has shown neuroprotective effects in rat models of hypoxic-ischemic encephalopathy (Moretto et al., 2005, 2009) and adult cortical focal ischemia, *via* multiple mechanisms including prevention of free radical attack and pro-inflammatory response (Hansel et al., 2014, 2015). Several other expression and function enhancers of EAAT2 are currently gathering attention as a potential therapeutic approach for a variety of adult disorders and await exploration in the context of the newborn brain (Fontana, 2015). It is currently unknown whether EAAT2 enhancers would restore glutamate uptake or exacerbate reverse transport in the preterm brain. Combination therapies targeting different mechanisms and therapeutic windows will also need exploring, including more established (i.e., magnesium sulfate) and more exploratory therapies (e.g., anti-inflammatory treatment) (Ofek-Shlomai and Berger, 2014).

Genetic risk stratification and pharmacogenomic approaches focusing on interindividual differences in treatment response are gathering interest and, as our healthcare systems develop, the integration of genomic data in clinical care seems an increasingly achievable goal (Rehm, 2017). Exploratory studies have implicated several functional genetic variants involved in glutamate excitotoxicity and inflammation in neurodevelopmental impairment, including as a sequelae of perinatal brain injuries (O’Callaghan et al., 2009, 2012, 2013; Wu et al., 2011; Kapitanovic Vidak et al., 2012). Among these, common genetic variants altering EAAT2 expression have been reported in association with cerebral palsy and neurodevelopmental delay in very preterm newborns (Rajatileka et al., 2017). Replication in larger samples, genome-wide designs and comparison with term brain injuries are needed to consolidate and expand the finding. Identification of panels of genetic variants that collectively increase risk of injury may be integrated with other types of clinical information and help identify high-risk pregnancies. Moreover, integration of genetic information has the potential to contribute to a more personalized approach to the care of the preterm newborn, with recent studies focusing on the interactions between genetic variants and responsiveness to antenatal magnesium sulfate therapy (Costantine et al., 2012; Clark et al., 2018). EAAT2 variants remain to be evaluated in this context.

Future *in vivo* studies will need to explore whether dysregulation of the main glutamate transporter, EAAT2, is central to the pathogenesis of preterm brain injuries or if it is a secondary process and whether the different cellular effects represent destructive or compensatory mechanisms. As explained by Danbolt (2001), “as long as one variable is not extreme, it will be the combination of several factors that will determine whether the ship will sink,” and several different primary events/changes may share a final common pathway. Well-designed animal model studies will be needed to provide mechanistic evidence. Human post-mortem studies can provide insights into patterns of dysregulation of expression, function, and localization specific to the different types of perinatal brain injuries, though limited by confounding factors, post-mortem artifacts, reproducibility, and sample size. Promising preliminary findings on the neuroprotective effects of EAAT2 suggest that this is certainly an avenue worth exploring.

## Author Contributions

KL and SP contributed to the conception and design of the review. SP wrote the first draft of the manuscript. All authors revised, read, and approved the submitted version of the manuscript.

### Conflict of Interest Statement

The authors declare that the research was conducted in the absence of any commercial or financial relationships that could be construed as a potential conflict of interest.
